# Supplementary material for: The Digital Divide in the Era of COVID-19: An Investigation into an Important Obstacle to the Access to the mHealth by the Citizen
Source: Healthcare (Basel). 2021 Mar 26;9(4):371. doi: 10.3390/healthcare9040371 (PMC8065806; doi:10.3390/healthcare9040371)
Supplement: Supplementary file 1 [file healthcare-09-00371-s001.pdf]

A study on the relationship with digital technology in the Covid-19 era

By dedicating us no more than a minute dedicated to the compilation you will give a big help to us and to a research on the difficulties of accessing digital technologies (in English the problem is called DIGITAL DIVIDE) Once completed: a) Help your friend / brother / parent / grandparent not familiar with digital technology to fill it out. Your answers will also be very useful to us. b) Help us by spreading it to friends and colleagues through social networks / messaging tools (eg. WhatsApp) and more

***** Obbligatoria

1. The data will be used anonymously for a study on this issue.

   ◯ I agree

   ◯ I don't agree

2. Sex *

   ◯ Male

   ◯ Female

   ◯ [ ]

   Altro

3. Age *

   Indicate the years completed

   [ ]

   Il valore deve essere un numero

4. ethnic groups in Italy *

They are indicated in italian

- Afroitaliano
- Albanesi
- Algerini in Italia
- Arbëreshë
- Austriaci
- Boliviani in Italia
- Bosniaci in Italia
- Brasiliani in Italia
- Britannici in Italia
- Bulgari in Italia
- Capoverdiani in Italia
- Catalani
- Cinesi in Italia
- Comunità armena di Venezia
- Comunità venetopontine
- Congolesi in Italia
- Croati in Italia
- Cubani in Italia
- Dominicani in Italia
- Ecuadoriani in Italia
- Egiziani in Italia
- Filippini in Italia
- Francesi in Italia

- Ghanesi in Italia
- Indiani in Italia
- Italiani
- Khorakhanè
- Kosovari in Italia
- Macedoni in Italia
- Moldavi in Italia
- Nepalesi in Italia
- Nigeriani in Italia
- Paraguaiani in Italia
- Peruviani in Italia
- Piemontákeri
- Polacchi in Italia
- Rom abruzzesi
- Rom in Italia
- Rom romeni in Italia
- Romeni in Italia
- Rumeni
- Sardi (etnia)
- Senegalesi in Italia
- Serbi di Trieste
- Serbi in Italia
- Sinti
- Sloveni
- Sloveni in Italia

- ◯ Somali in Italia
- ◯ Spagnoli in Italia
- ◯ Srilankesi in Italia
- ◯ Sudtirolesi
- ◯ Svizzeri in Italia
- ◯ Tedeschi
- ◯ Tedeschi in Italia
- ◯ Togolesi in Italia
- ◯ Tunisini in Italia
- ◯ Turchi in Italia
- ◯ Ucraini in Italia
- ◯ Uruguaiani in Italia
- ◯ Walser
- ◯ ____________ Altro

5. Enter your qualification *

indicate your highest title held

.

- Primary school diploma
- middle School diploma
- high school diploma
- first degree
- master's degree
- doctorate
- university specialization
- first level master
- second level master
- Altro

6. Do you work at the moment? *

- Yes
- No

7. What sector do you work in? *

Choose the most relevant answer

.

- ○ Public (employee or other forms of subordination)
- ○ Private (employee or other forms of subordination)
- ○ Trade and / or craft entrepreneurship
- ○ Of the liberal professions (eg. Engineer, Lawyer, etc.)
- ○ [ ] Altro

8. Add more information about your job description *

[ ]

9. .In which region / autonomous province do you work? *

- ◯ Abruzzo
- ◯ Basilicata
- ◯ Calabria
- ◯ Campania
- ◯ Emilia Romagna
- ◯ Friuli Venezia Giulia
- ◯ Lazio
- ◯ Liguria
- ◯ Lombardia
- ◯ Marche
- ◯ Molise
- ◯ Piemonte
- ◯ Provincia Autonoma di Bolzano
- ◯ Provincia Autonoma di Trento
- ◯ Puglia
- ◯ Sardegna
- ◯ Sicilia
- ◯ Toscana
- ◯ Umbria
- ◯ Valle d'Aosta
- ◯ Veneto

10. Your region / autonomous province at the moment by the recent DPCM based on the criteria issued has been defined in the following area *

- ○ Red
- ○ Jellow
- ○ Orange
- ○ [ ] Altro

11. In which region / autonomous province are you currently domiciled? *

- ◯ Abruzzo
- ◯ Basilicata
- ◯ Calabria
- ◯ Campania
- ◯ Emilia Romagna
- ◯ Friuli Venezia Giulia
- ◯ Lazio
- ◯ Liguria
- ◯ Lombardia
- ◯ Marche
- ◯ Molise
- ◯ Piemonte
- ◯ Provincia Autonoma di Bolzano
- ◯ Provincia Autonoma di Trento
- ◯ Puglia
- ◯ Sardegna
- ◯ Sicilia
- ◯ Toscana
- ◯ Umbria
- ◯ Valle d'Aosta
- ◯ Veneto

12. Indicate your municipality of residence *

13. Which of the following tools are you familiar with? *

indicate even more answers

- [ ] Tablet
- [ ] laptop computer
- [ ] smartphone
- [ ] computer
- [ ] Altro

14. Do you have an adequate Internet connection (fixed or mobile)? *

- ( ) Yes
- ( ) No

15. Do you have one or more smartphones?

. *

- ( ) Yes
- ( ) No

16. Indicate your degree of familiarity with social network apps (eg Facebook) *

	1	2	3	4	5	6	
none	( )	( )	( )	( )	( )	( )	a lot

17. Indicate your degree of familiarity with messaging apps (eg. WhatsApp) *

	1	2	3	4	5	6	
none	○	○	○	○	○	○	a lot

18. Indicate your degree of familiarity with Immuni (Contact tracing app) *

	1	2	3	4	5	6	
none	○	○	○	○	○	○	a lot

19. Indicate your degree of familiarity with other Apps *

please do not consider the medical Apps (MA) here. For the MA there is a specific question in the following)

	1	2	3	4	5	6	
none	○	○	○	○	○	○	a lot

20. Indicate your degree of familiarity with the use of mobile devices for medical and health practices *

	1	2	3	4	5	6	
none	○	○	○	○	○	○	a lot

21. indicate your degree of familiarity with the use of digital and IT tools for medical and health practices *

	1	2	3	4	5	6	
none	○	○	○	○	○	○	a lot

22. Indicate if you have a disability or other problems that do not allow you to use IT tools independently *

- ◯ Yes
- ◯ No

23. Indicate if in this period you have been involved in remote visit / care / diagnosis processes through digital technologies *

- ◯ Yes
- ◯ No

24. Can you tell us something *

25. Add a final comment if you wish

Questo contenuto non è stato creato né approvato da Microsoft. I dati che invii verranno recapitati al proprietario del modulo.

Microsoft Forms
